# Effects of Low Temperature at Booting Stage on Sucrose Metabolism and Endogenous Hormone Contents in Winter Wheat Spikelet

**DOI:** 10.3389/fpls.2019.00498

**Published:** 2019-04-18

**Authors:** Wenjing Zhang, Jiaqin Wang, Zhenglai Huang, Lu Mi, Kaifang Xu, Jiajia Wu, Yonghui Fan, Shangyu Ma, Dongguo Jiang

**Affiliations:** Department of Agronomy, Key Laboratory of Wheat Biology and Genetic Improvement on South Yellow and Huai River Valley, the Ministry of Agriculture, Anhui Agricultural University, Hefei, China

**Keywords:** wheat, spring low temperature, booting stage, sucrose metabolism, hormone content, yield

## Abstract

Low spring temperatures often occur during the winter wheat booting stage, when the young ears are very sensitive to cold. In this study, we used two wheat varieties differing in cold sensitivity (sensitive variety Yangmai 18 and tolerant variety Yannong 19) to examine the effect of low temperature on wheat grain number at booting stage. Low temperature stress was simulated in an artificial climate chamber at 4°C for 60 h in 2016 and at 2, 0, or −2°C for 24 h in morphological assays, showing that the development of wheat spikelets was inhibited and floret growth was delayed following low temperature stress. However, an increase in the sucrose content of young panicles was also observed, and the activity of enzymes involved in sucrose metabolism was dynamically altered. Sucrose phosphate synthase activity was enhanced, and sucrose synthase activity significantly increased after treatment at 4 and 2°C, respectively. However, activities of sucrose synthase and invertase decreased with a reduction in temperature. Gene expression assays further revealed downregulation of *TaSuS1* expression and upregulation of *TaSuS2*, while expression of *CWINV* was inhibited. Moreover, phytohormone content assays showed an increase in the content of abscisic acid in young wheat ears, but a decrease in the content of auxin and gibberellins. The grain number per spike and 1000-grain weight also showed a downward trend following low temperature stress. Overall, these findings suggest that low temperature at booting induces abscisic acid accumulation in winter wheat, altering the activity of the enzymes involved in sucrose metabolism, which leads to an accumulation of sucrose in the young ears, thereby having a negative effect on wheat production.

## Introduction

Extreme climate events have increased significantly. As a result, low temperature in spring has become a major constraint of winter wheat production, especially in southern Huanghuai and the middle and lower reaches of the Yangtze River, China. Moreover, the increase in global average winter temperatures promotes winter growth, increasing cold vulnerability in the spring (Li et al., 2016). Low temperatures in spring generally occur between the end of March and beginning of April, during which time wheat ears are in a critical period of meiosis and tetrad formation and are, therefore, highly sensitive to temperature stress. If exposed to low temperature at this time, the entire wheat spike or parts of the spikelet will fail to set after tassel formation, decreasing yield by 30–50% (Zhang et al., 2011).

From booting to anthesis, significant deterioration in the wheat florets occurs, and pollen development enters the meiosis stage, and the most cold-sensitive period (Slafer et al., 2001). Low temperatures induce abnormal pollen and ovule development, resulting in a reduction in anther size and fertile pollen grains, thereby decreasing the rate of seed setting (Thakura et al., 2010). In rice, cold damage during the reproductive stage results in spikelet degradation and an increase in sterile spikelets (Wang et al., 2013). In the early stages of pollen development, anthers are the strongest metabolic pool, and large amounts of sugars are therefore transported to the anthers to support development (Castro and Clément, 2007). Serrago et al. (2008) revealed a positive correlation between basal spikelet gestation floret and the dry weight, as well as carbohydrate content at flowering, suggesting that the supply of assimilates determines the number of fertile florets.

Abnormal glucose metabolism, a lack of starch accumulation in the pollen grains and abnormal degradation of the tapetum are thought to be the main causes of floret abortion (Ji et al., 2013; Niu et al., 2013). Low temperature stress also has a significant effect on sugar metabolism (Wanner and Junttila, 1999). In order to enhance membrane stability, plants accumulate a large amount of carbohydrates under low temperature, inducing stomatal closure, photosynthetic electron transport damage and inhibiting carbon assimilation (Li et al., 2014). Low temperature stress also reduces the supply of soluble carbohydrates to reproductive tissues, leading to nutrient deficiencies in the tapetum and endosperm (Nayyar et al., 2005). Sucrose is the main form of sugar transported via the phloem. Sucrose phosphate synthase (SPS, EC 2.4.1.14) is the key enzyme involved in regulating sucrose synthesis (Sharma et al., 2010), while sucrose synthase (SuS, EC 2.4.1.13) plays a major role in catalyzing sucrose degradation in plant tissues. Invertase (Inv, EC 3.2.1.26) degrades sucrose into fructose and glucose via acid invertase (AI) or neutral invertase (NI) depending on the pH. In rice pollen grains, AI activity is inhibited under low temperature stress, and therefore, the amount of sucrose transported to the tapetum and pollen grains decreases, resulting in pollen abortion (Oliver et al., 2005).

Plant endogenous hormones act as signaling factors during low temperature stress, with abscisic acid (ABA) and gibberellic acid (GA) playing an important role in cold resistance (Sun et al., 2009). Increased endogenous ABA levels induce upregulation of stress response genes, thereby enhancing cold tolerance (Yang et al., 2011). Oliver et al. (2007) further revealed that low temperature stress induced an increase in ABA accumulation in rice, causing a decrease in Inv activity in the cell wall of the tapetum, which is the main cause of pollen abortion. GA also plays an important role in initiating meiosis and development of the tapetum in pollen mother cells. In rice pollen, for example, high temperature stress was found to cause a decrease in GA and a significant increase in the floret abortion rate (Tang et al., 2008).

A series of studies has shown that the photosynthetic rate of functional wheat leaves decreases significantly under low temperature stress at booting (Zhang et al., 2015, 2016), with an accumulation of carbohydrates (Zeng et al., 2011) and changes in endogenous hormone contents and antioxidant enzyme activity (Sun et al., 2009; Li et al., 2014). However, few studies have documented the effects of low temperature at booting on young wheat ears. In this study, two wheat varieties differing in cold sensitivity were selected to determine the effect of low temperature at booting on sucrose metabolism and hormone contents in young wheat ears. Physiological causes for the resulting effects and the subsequent decrease in ear grain number are also discussed.

## Materials and Methods

### Experimental Design

This study was conducted at the Anhui Agricultural University, Anhui, Hefei, China, from October 2015 to June 2017 (31.52°N, 117.17°E). Two wheat varieties differing in cold sensitivity were selected: cold-tolerant Yannong 19 and cold-sensitive Yangmai 18. Plants were placed in pots 28 cm in height and 30 cm in diameter. Potting soil was obtained from the 0–20 cm tillage layer and had an organic matter content of 15.80 g kg^−1^, a total nitrogen content of 0.78 g kg^−1^, and available nitrogen, available phosphorus and available potassium contents of 104.9, 22.8, and 150.5 mg kg^−1^, respectively. The soil was sieved, then 8 kg was placed in each pot. Wheat seeds were sowed on October 31, 2015 and October 28, 2016. Before planting, 75 g of organic fertilizer, 1.05 g of pure nitrogen, 1.25 g of P_2_O_5_ and 2.25 g of K_2_O were applied to each pot, with an additional 1.05 g of pure nitrogen applied at the jointing stage. The pots were then buried with the soil surface flush to ground level in the experimental plots. Other management measures were carried out in accordance with the requirements for high-yield cultivation.

On April 2, 2016 and April 3, 2017, when the young ears reached the meiosis stage, 60 pots per variety were placed in an artificial climate chamber. In 2016, the temperature in the chamber was maintained at 4°C, and the plots were moved back to the field after 60 h treatment. In 2017, the temperature in the chamber was set at −2, 0, or 2°C between 19:00 and 07:00 and at 5°C between 07:00 and 19:00, and the pots were moved back to the field after 24 h of treatment. Humidity in the chamber was kept at 70% in both years. The wheat plants were then kept in the field until maturity.

### Sampling

At the end of each treatment, 10 young ears of similar size were sampled. Three were used for morphological observations, and the remainder were frozen in liquid nitrogen and stored at −40°C for analysis of sucrose content, enzyme activity and endogenous hormone content. The spikelet number per ear of both varieties is generally about 18–20. In order to analyze the development between spikelets in different locations, the middle 6 spikelets are called middle spikelets, which are expressed by MS, and the upper 6 to 7 spikelets are expressed by US. The lower 6 to 7 spikelets are represented by LS. In this study, the young ear represents the entire ear, including US, MS, and LS. Yield traits including grain number per spike and the 1000-grain weight were measured after harvest. Untreated plants were used as controls.

### Observation of Floret Morphology

Fresh wheat spikelets were sampled from control and low temperature treatment. One to three basal florets were removed from each spikelet, then the images were taken with a SZX16 stereo microscope (OLYMPUS, Japan).

### Measurements of Sucrose Content

The young ears were incubated at 105°C for 15 min then dried at 70°C to a constant weight. Four spikelets from each of US, MS, and LS were ground into a powder, then 0.5 g samples were placed in a 10 ml centrifuge tube, to which 4 ml of 80% ethanol was added. The tubes were then incubated at 80°C for 40 min in a water bath with shaking before centrifuging at 4000 rpm for 5 min. The supernatant was collected, then 2 ml of 80% ethanol was added to the residue for re-extraction. The resulting supernatants were combined, then 10 mg of activated carbon was added and decolorization at 80°C for 30 min was carried out. The reaction mixture was made up to 10 ml with 80% ethanol. After filtration, the obtained filtrate was used to determine the sucrose content as described by Hendrix (1993).

### SPS, SuS, and Inv Enzyme Activity

Fresh spikelet samples weighing 1.0 g were mixed with 10 ml of Hepes-NaOH buffer (pH 7.5). After grinding on ice, the samples were centrifuged at 10,000 rpm for 10 min at 4°C, and the resulting supernatant was used to determine activities of SPS, SuS, and Inv as described below.

Sucrose phosphate synthase activity was determined according to the method of Zhang et al. (2009). Briefly, 0.5 ml of crude enzyme solution was mixed with a reaction solution consisting of 0.1 ml 50 mM fructose-6-phosphate, 0.1 ml 50 mM UDPG, 0.1 ml Hepes-NaOH buffer, and 0.05 ml 10 mM MgCl_2_, then maintained at 30°C for 30 min. To terminate the reaction, 200 μl of 40% NaOH was added, followed by 1.5 ml of 30% HCL and 0.5 ml of 1% resorcinol. The sucrose content was then determined by measuring the absorbance at 480 nm using a UV-8000 double beam ultraviolet visible spectrophotometer (Shanghai Metash Instruments Co., Ltd., Shanghai, China). SuS activity was determined using the same method except that fructose was substituted for fructose 6-phosphate.

Acid invertase activity was measured as described by Shu et al. (2009). Briefly, the extraction mixture was incubated at 45°C for 1 h, then 1 ml of solution obtained before and after transformation was added to a 25-ml volumetric bottle, to which 2 ml of 3,5-dinitrosalicylic acid solution (DNS) was added. The color was allowed to develop in a boiling water bath for 3 min; then, after cooling, the volume was increased to 25 mL, and the resulting glucose content was determined by the absorbance at 540 nm.

### Quantitative Assays of *TaSUS1*, *TaSUS2*, and *TaCWI* Expression

To determine expression levels of the genes involved in sucrose metabolism under cold stress, three genes, *TaSuS1*, *TaSuS2*, and *TaCWI*, were selected (Oliver et al., 2005; Ji et al., 2011). Gene-specific primers were designed according to the cDNA gene sequences published in the NCBI and synthesized by Sangon Biotechnology Co., Ltd. (Shanghai, China). Primer sequences are listed in Table 1. Total RNA was extracted using Trizol (Invitrogen, Carlsbad, CA, United States, SK1321) according to the manufacturer’s instructions. Reverse transcription was then carried out using a cDNA synthesis kit (Thermo Fisher Scientific^TM^, EP0733). Gene transcription levels in the wheat ears were determined using an ABI Prism 7500 Real-Time PCR System (Applied Biosystems, Foster City, CA, United States) and SG Fast qPCR Master Mix (2X) (Roche, B639271). PCR was carried out as follows: pre-denaturation at 95°C for 3 min followed by 45 cycles of denaturation at 95°C for 7 s, annealing at 57°C for 10 s, and extension at 72°C for 15 s. Relative expression levels of the target genes were calculated using the 2^−ΔΔCt^ method (Livak and Schmittgen, 2001).

**Table 1:** Primers used to detect differential gene expression using quantitative PCR.

Gene	Primer sequence (5′–3′)	Production size (bp)
*TaSuS1*	F: TGAAGTGTCGGCTGCGTTAT	162
	R: ATGGGCAGGCGTTTATTCC	
*TaSuS2*	F: CCGAGCCACTGGAACAAGAT	174
	R: GGCGTAGAGCATTTCAAGGTAG	
*TaCWI*	F: GGACCTGCATCCTATCGAGAGT	110
	R: TGCCGTTAGTTTGGACACCTT	
18s	F: TTAACGAACGAGACCTCAGCC	
	R: TGCCGTTAGTTTGGACACCT	

### Measurements of Endogenous Phytohormone Contents

Endogenous hormone contents were determined by enzyme-linked immunosorbent assay (ELISA) as described by Han et al. (2015). Fresh wheat spikelets (0.5 g) were ground into a homogenate on ice using extraction buffer (80% methanol containing 1 mM di-tert-butyl-p-cresol BHT). The obtained samples were then transferred to a 10 ml centrifuge tube and incubated at 4°C in a shaker overnight. The mortar was then rinsed with 3 ml of extract, transferred to a test tube, shaken, then placed in a refrigerator at 4°C overnight. The samples were then centrifuged at 3500 rpm for 8 min and the supernatant was filtrated with a C-18 solid phase extraction column. The resulting sample was transferred to a small 25 ml beaker and the methanol in the extract removed by vacuum freeze drying. The final volume was then adjusted to 1 ml with sample dilution buffer. All kits were provided by China Agricultural University.

### Measurements of Grain Number per Spike and 1000-Grain Weight

After reaching maturation, 20 pots were randomly selected from the remaining unsampled pots and used to calculate the grain number per spike and 1000-grain weight. Both measurements were repeated in triplicate.

### Statistical Analysis

All data presented represent the mean value ± SE of three independent duplications. Data were subjected to analysis of variance (ANOVA), and statistical divergence among treatments was determined using Duncan’s multiple range test (*P* < 0.05). Coefficients and *p*-values of the correlations were calculated using Pearson’s correlation coefficient. Statistical analyses were conducted using SPSS statistical software (version 10; SPSS, Inc., Chicago, IL, United States).

## Results

### Young Ear and Floret Morphology

**FIGURE 1:**
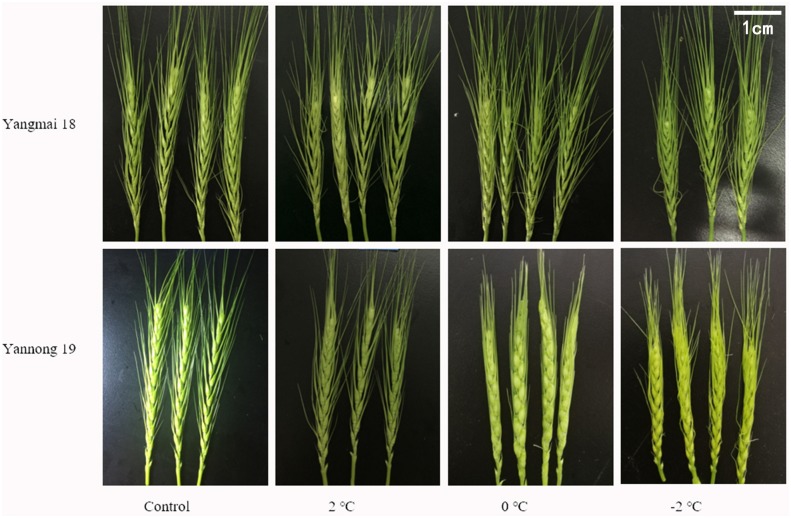
Effect of low temperature on the morphology of wheat ears at booting stage in 2017. Wheat plants (cold-sensitive variety Yangmai 18 and more tolerant variety Yannong 19) were placed under 2, 0, or –2°C for 24 h, then morphological observations of the young ears were made. As a control, plants were grown without cold stress.

Low temperature (LT) stress at booting had multiple effects on the wheat spikelets and florets (Figure 1). The untreated control ears were full, and the awns were firm and upright; however, in contrast, the young ears were smaller, and the top and basal spikelets were not fully developed after LT treatment. The wheat awns were also deformed and uneven. The smallest ears were observed after treatment at −2°C. Overall, these findings suggest that LT stress at booting hinders the development of wheat spikelets.

**FIGURE 2:**
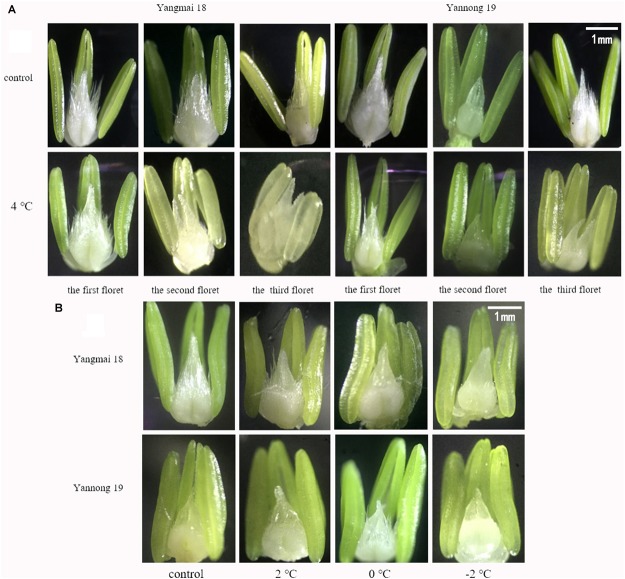
Effect of low temperature on the morphology of wheat florets at booting stage in 2016 and 2017 (×4 magnification). Wheat plants were placed under 4°C for 60 h. Wheat plants were placed under 2, 0, or –2°C for 24 h, then morphological observations of the florets were made. As a control, plants were grown without cold stress. **(A)** Florets in the middle spikelet. **(B)** First florets in the middle spikelet.

After treatment at 4°C for 60 h, the first floret of the MS was less affected than the second and third florets in Yangmai 18, while in Yannong 19 the third floret was more affected (Figure 2A). Moreover, floret development was significantly delayed in both varieties relative to the controls. Treatment at 2°C for 24 h had less effect on the first floret of the MS; however, after 0°C treatment, a decrease in floret volume was observed and development was slower compared to the controls. Similarly, after −2°C treatment, floret development was slow, and they were lighter in color compared to the controls (Figure 2B). These findings suggest that LT stress inhibited development of the florets.

### Increased Accumulation of Sucrose in Ears of the Cold Tolerant Variety

**FIGURE 3:**
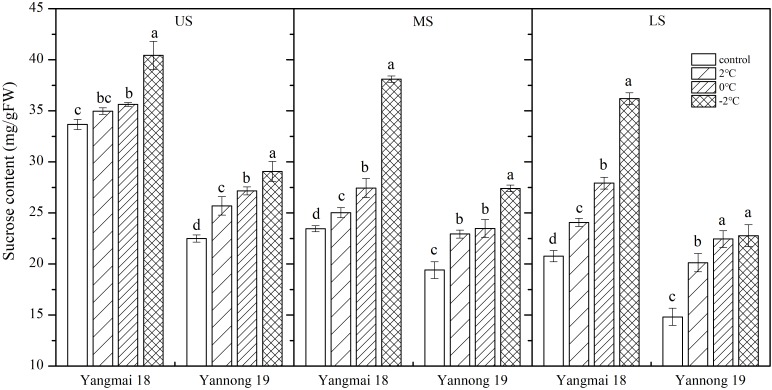
Effect of low temperature on the sucrose content of wheat ears at booting stage in 2017. US, upper spikelets; MS, middle spikelets; LS, lower spikelets. Wheat plants were placed at 2, 0, or –2°C for 24 h, respectively. As a control, plants were grown without cold stress. Data represent means ± SE (*n* = 3). Bars not sharing the same lowercase letter are significantly different according to Duncan’s multiple range test (*P* < 0.05).

Low temperature stress at booting increased in the sucrose content of the wheat spikes, and this increase was greater with decreasing temperature (Figure 3). In spikelets of cold-sensitive Yangmai 18, a significant increase in sucrose content was observed at all temperatures compared with the control, while the in cold-tolerant variety Yannong 19 a significant difference was observed in the US only at all temperatures (*P* < 0.05). The differences in sucrose content among spikelet locations were also significant (Table 2). In the US of Yangmai 18, increases of 15.90, 34.49, and 74.36% were observed, after 2, 0, and −2°C treatment for 24 h, respectively. Meanwhile, in the MS and LS, increases of 6.66, 16.94, and 62.37% as well as 3.87, 5.80, and 20.10% were observed, respectively. In the US of Yannong 19, increases in sucrose content of 35.91, 51.54, and 53.71% were observed, after 2, 0, −2°C treatment for 24 h, respectively. Meanwhile, in MS and LS increases of 18.17, 20.99, and 41.23 %, and 14.22, 20.76, and 29.21 % were observed, respectively. These findings suggest that the increase in sucrose content was greater in young ears of Yannong 19 than those of Yangmai 18 (Table 2).

### Sucrose Metabolism-Related Enzyme Activity

#### Increased SPS Activity After LT Stress

**FIGURE 4:**
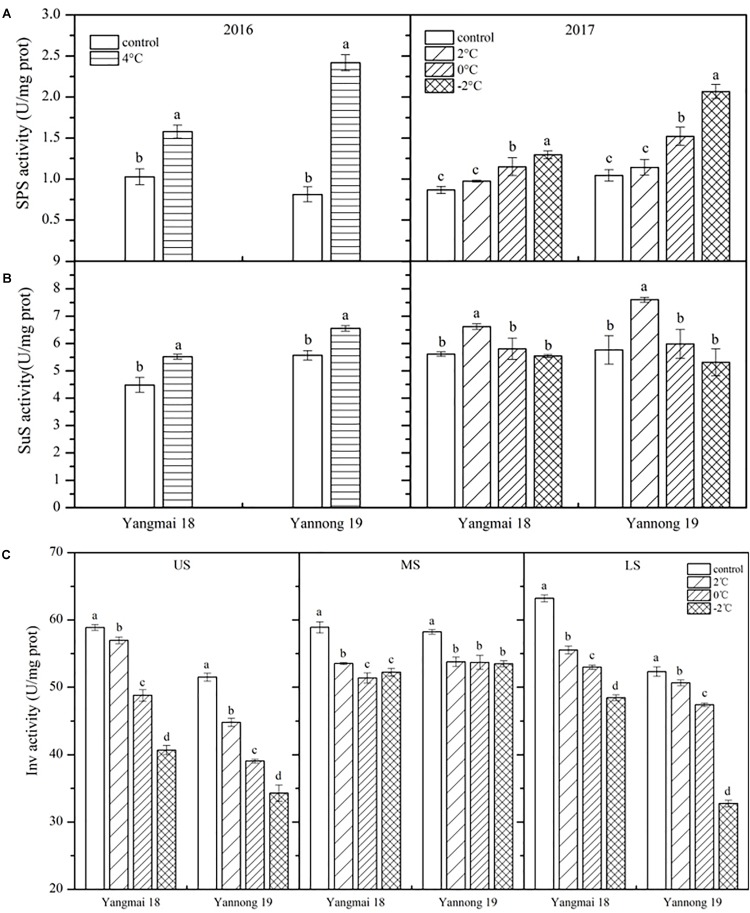
Effect of low temperature on the activities of sucrose phosphate synthase **(A)**, sucrose synthase **(B)**, and invertase **(C)** in wheat ears at booting stage in 2016 and 2017. US, upper spikelets; MS, middle spikelets; LS, lower spikelets. Wheat plants were placed under 4°C for 60 h in 2016 and at 2, 0, or –2°C for 24 h in 2017. Data represent means ± SE (*n* = 3). Bars not sharing the same lowercase letter are significantly different according to Duncan’s multiple range test (*P* < 0.05).

As shown in Figure 4A, SPS activity was enhanced in the wheat spikes following LT stress, and this increase increased with reducing temperature. Significant differences were observed between treated and control plants of both varieties after 4°C treatment for 60 h and at 0 and −2°C treatment for 24 h (*P* < 0.05). However, no significant differences in SPS activity were observed after treatment at 2°C. In Yangmai 18, SPS activity increased by 53.38% after treatment at 4°C for 60 h, while in Yannong 19 an increase of 197.03% was observed. After treatment at 2, 0, and −2°C for 24 h, SPS activity increased by 12.28, 32.62, and 49.30% in Yangmai 18, respectively, while in Yannong 19 increases of 9.35, 45.45, and 97.66% were observed. Overall, the increase in SPS activity was greater in young ears of Yannong 19 than those of Yangmai 18 under the same LT treatment (Figure 4A and Table 2).

#### SuS Activity Fluctuated With Temperature

The effects of LT stress at booting on SuS activity varied between treatments (Figure 4B). After treatment at 4°C for 60 h and 2°C for 24 h, SuS activity increased in spike of both varieties, and the differences between treatments and controls were significant (*P* < 0.05). Meanwhile, after 0 and −2°C treatment for 24 h, SuS activity decreased compared to 2°C treatment in both varieties, while at 0°C, activity was higher than that of the controls by 3.42 and 3.78%, respectively, in Yangmai 18 and Yannong 19. In contrast, following treatment at −2°C, SuS activity decreased in both varieties compared to the control by 1.32 and 7.91%, respectively. Despite these findings, none of the differences in SuS activity between varieties and among treatments were significant (Table 2).

#### LT Stress Decreased Inv Activity

Low temperature stress at booting caused a decrease in Inv activity, and this decrease increased with temperature (Figure 4C). Significant differences in Inv activity were observed among treatments and spikelet locations (*P* < 0.05, Table 2). In Yangmai 18, treatment at 2, 0, and −2°C for 24 h caused reductions in Inv activity in the US of 3.22, 3.97, and 30.90%, respectively, while in the MS and LS, decreases of 9.13, 12.79, and 11.36%, and 12.17, 16.19, and 23.35% were observed, respectively. In Yannong 19, treatment at 2, 0, and −2°C for 24 h caused reductions in Inv activity in the US of 13.02, 24.17, and 33.41%, respectively, while in the MS and LS, decreases of 7.64, 7.77, and 8.14%, and 3.17, 9.38, and 37.38% were observed, respectively. Moreover, a significant decrease in Inv activity with decreasing temperature was observed in the US and LS compared with the MS (Figure 4C and Table 2).

### Expression of Genes Implicated in Sucrose Metabolism

**FIGURE 5:**
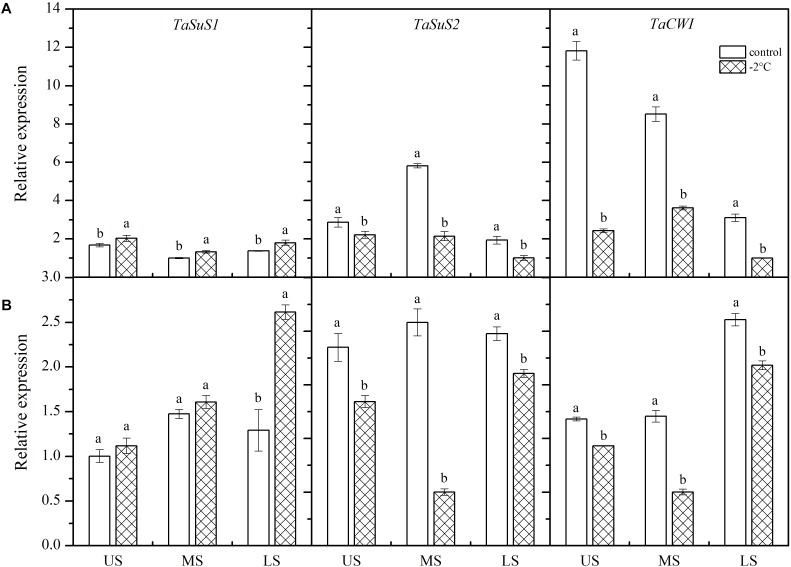
Effect of low temperature on the relative expression of *TaSus1*, *TaSus2*, and *TaCWI* in wheat ears of Yangmai 18 **(A)** and Yannong 19 **(B)** plants in 2017. US, upper spikelets; MS, middle spikelets; LS, lower spikelets. Wheat plants were placed under –2°C for 24 h. As a control, plants were grown without cold stress. Data represent means ± SE (*n* = 3). Bars not sharing the same lowercase letter are significantly different according to Duncan’s multiple range test (*P* < 0.05).

After treatment at −2°C for 24 h, *TaSUS1* expression in the wheat ears was upregulated, while expression of *TaSUS2* and *TaCWI* was downregulated (Figure 5). In Yangmai 18 (Figure 5A), significant differences in *TaSUS1* expression were observed in different parts of the young ears compared with the control (*P* < 0.05). However, in Yannong 19 (Figure 5B), only expression of *TaSUS1* in the LS was significantly different than the control, with a 2.02-fold increase. Expression of *TaSUS2* in the MS and LS was significantly different from that of the controls in both varieties, with decreases of 59.99 and 76.06% in Yangmai 18 and Yannong 19, respectively. Expression of *TaCWI* was significantly reduced in Yangmai 18 compared to Yannong 19 after LT stress. The expression of *TaCWI* was significantly reduced in Yangmai 18 compared to Yannong 19 after LT stress.

### Effect of LT Stress on the Hormone Contents of the Wheat Ears

#### LT Stress Induced the ABA Content

**FIGURE 6:**
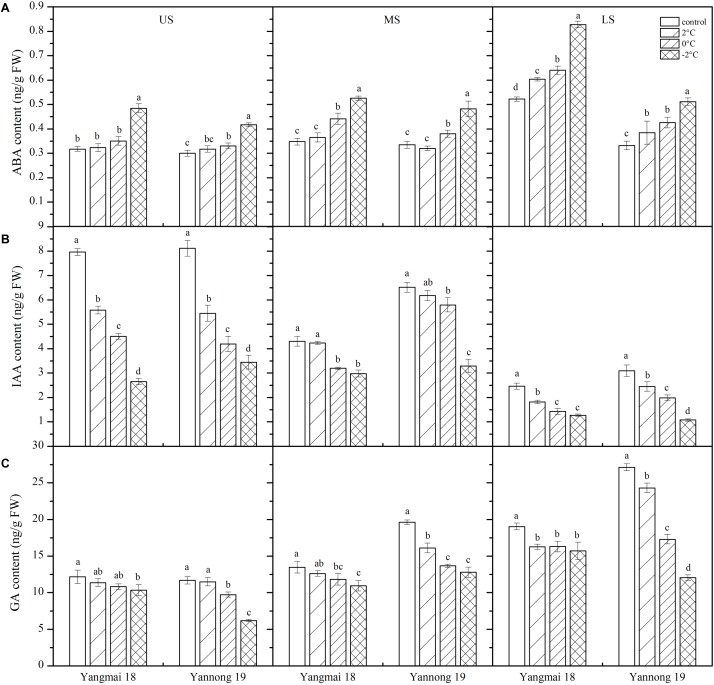
Effect of low temperature on the contents of abscisic acid **(A)**, auxin **(B)**, and gibberellin **(C)** in wheat ears at booting stage in 2016 and 2017. US, upper spikelets; MS, middle spikelets; LS, lower spikelets. Wheat plants were placed under 2, 0, or –2°C for 24 h. As a control, plants were grown without cold stress. Data represent means ± SE (*n* = 3). Bars not sharing the same lowercase letter are significantly different according to Duncan’s multiple range test (*P* < 0.05).

Abscisic acid plays an important role in the response to LT stress (Mittler and Blumwald, 2015). In this study, LT stress at booting caused an increase in ABA content in the wheat ears, except in the MS of Yannong 19, in which the ABA content decreased slightly (Figure 6A). Significant differences in ABA content were observed among temperature treatments and spikelet locations (Table 2). After treatment at 2°C for 24 h, the ABA content in the US, MS, and LS of Yangmai 18 increased by 1.78, 4.98, and 15.66%, respectively, while in Yannong 19 increases of 5.72 and 15.94% were observed in the US and LS, respectively. After −2°C treatment for 24 h, the ABA contents in the US, MS, and LS of Yangmai 18 increased by 52.48, 51.21, and 58.57%, respectively, while in Yannong 19 increases of 38.98, 43.94, and 54.20% were observed, respectively. Moreover, a significant increase in ABA content was observed in the LS compared to the MS and US. In the US and MS, the ABA content increased significantly after −2°C treatment for 24 h. Moreover, the ABA content in the Yannong 19 spikelets remained at a relatively low level compared with Yangmai 18 (Figure 6A and Table 2).

#### LT Stress Reduced the IAA Content

Low temperature stress at booting caused a decrease in IAA content in the spikelets of both varieties (Figure 6B) and the differences among temperature treatments and spikelet locations were all significant (Table 2). Taking Yangmai 18 as an example, treatment at 2°C for 24 h caused a decrease in IAA content in the US, MS, and LS of 29.89, 1.63, and 26.24%, respectively. Meanwhile, treatment at 0°C and −2°C for 24 h caused decreases of 43.3, 25.76, and 42.15%, and 66.66, 30.73, and 48.39%, respectively. A significant decrease in the IAA content in the US and LS was therefore observed compared to that in the MS (Table 2). Overall, the IAA content was higher in the Yannong 19 spikelets than in Yangmai 18 under all temperature treatments (Figure 6B and Table 2).

#### LT Stress Reduced the GA Content

Inhibition of endogenous growth-promoting GA signaling pathways significantly increases plant survival under stress (Achard et al., 2006). In this study, LT treatment at booting caused a decrease in GA content in the spikes of both varieties (Figure 6C), and the differences among temperature treatments and spikelet locations were all significant (Table 2). Taking Yannong 19 as an example, treatment at 2°C for 24 h caused decreases in GA content in the US, MS and LS of 1.59, 17.93, and 10.42%, respectively. Meanwhile, treatment at 0°C and −2°C for 24 h caused decreases of 16.66, 30.32, and 36.26, and 46.97, 34.83, and 55.65%, respectively. Overall, a significant decrease in GA contents was observed in the US and LS compared with the MS, and the content was higher in the Yannong 19 spikelets than in Yangmai 18 under all temperature treatments (Figure 6C and Table 2).

### LT Stress Reduced Grain Yield

As shown in Table 3, LT stress caused a decrease in the final grain number and 1000-grain weight. After treatment at 4°C for 60 h, a significant difference in grain number and 1000-grain weight was observed between LT treated plants and controls (*P* < 0.05). In contrast, treatment at 2°C and 0°C for 24 h caused no significant differences in the number of grains per ear. However, further reductions in temperature to −2°C for 24 h caused a significant difference in the number of grains per ear compared with the control. Moreover, the 1000-grain weights of both Yangmai 18 and Yannong 19 were significantly reduced compared to the controls.

**Table 2:** ANOVA of enzyme activities and hormone contents relative to cultivar, temperature treatment, spikelet location, and cultivar+temperature/cultivar+temperature+location.

		SC	SPS	SuS	Inv	ABA	IAA	GA
	Source of variation							
	C	nd	^∗∗^	^∗∗^	nd	nd	nd	nd
2015–2016	T		^∗∗^	^∗∗^				
	L							
	C × T/C × T × L		^∗∗^	ns				
								
	C	^∗∗^	^∗∗^	ns	^∗∗^	^∗∗^	^∗∗^	^∗∗^
2016–2017	T	^∗∗^	^∗∗^	^∗∗^	^∗∗^	^∗∗^	^∗∗^	^∗∗^
	L	^∗∗^			^∗∗^	^∗∗^	^∗∗^	^∗∗^
	C × T/C × T × L	^∗∗^	^∗∗^	ns	^∗∗^	^∗∗^	^∗∗^	^∗∗^

*SC, sucrose content (mg/g FW); SPS, phosphosucrose synthase activity (U/mg pro); SuS, sucrose synthase activity (U/mg pro); Inv, invertase activity (U/mg pro); ABA, abscisic acid content (ng/g FW); IAA, indoleacetic acid content (ng/g FW); GA, gibberellic acid content (ng/g FW). nd, no data; ns, not significant; ^∗∗^Significant differences at 0.01.*

Analysis of the relationships between yield factors and sucrose metabolism-related enzyme activity and hormone content revealed a negative correlation between sucrose content, SPS activity and ABA content and grain number per ear and the 1000-grain weight following LT stress (Table 4). Positive correlations between yield factor and SuS activity, Inv activity, IAA content and GA content were also observed as well as significant negative correlations (*P* < 0.05) between grain number per ear, the 1000-grain weight and sucrose and ABA content. Significant negative correlations were also observed between 1000-grain weight and Inv activity, IAA content and GA content in Yangmai 18. A significant negative correlation between 1000-grain weight and sucrose content, and a significant negative correlation between grain number per ear and ABA content was observed in Yannong 19.

## Discussion

Jointing to booting are key stages in terms of the differentiation and development of wheat spikes. At this time, LT stress inhibits the supply of sucrose and limits the development of pollen (Oliver et al., 2005), resulting in floret abortion and a decrease in the seed setting rate (Thakura et al., 2010).

### Effects of LT at Booting on Sucrose Metabolism in Wheat Ears

Carbohydrate metabolism is not only crucial to plant development but is also an essential component of the response to abiotic stress. Serrago et al. (2008) found a positive correlation between the number of spikelets, number of grains and dry weight and the carbohydrate content at flowering. In the early stage of microspore development, anthers are the most important component of the flower, and a large amount of sugar is therefore transported to the anthers to support the development of microspores (Wang et al., 2013). LT stress reduces the supply of soluble carbohydrates to the reproductive tissues, leading to nutrient deficiencies in the tapetum and endosperm (Nayyar et al., 2005). It is generally believed that abnormal sucrose metabolism under LT stress, a lack of starch accumulation in the pollen grains and abnormal degradation of the tapetum are the main causes of floret abortion (Ji et al., 2013; Niu et al., 2013). The accumulation of sucrose plays an essential role in cold adaptation in wheat (Zeng et al., 2011). Moreover, Sakata et al. (2014) found that application of sucrose greatly improved the fertility of rice pollen under LT stress, increasing the seed setting rate. In this study, we found that sucrose accumulation in the wheat spikelets continued to increase under LT stress at booting, and moreover, this increase increased with decreasing temperature, especially in the MS and LS. These findings suggest that sucrose accumulation helps wheat adapt to cold stress.

**Table 3:** Grain number and grain weight of Yangmai-18 and Yannong-19 after low temperature treatment at booting stage in the 2015–2017 growing seasons.

Year	Cultivar	Treatment	Grain number per ear	1000-grain weight (g)
	Yangmai 18	control	47.60a ± 1.52	45.09a ± 0.21
		4°C	45.00b ± 1.00	39.66b ± 0.59
	Yannong 19	control	42.40a ± 0.89	39.02a ± 0.64
2015–2016		4°C	38.40b ± 1.52	35.82b ± 0.52
	*F*-value	*F*-_Cultivar (C)_	136.51^∗∗^	147.745^∗∗^
		*F*-_Treatment (T)_	42.706^∗∗^	104.068^∗∗^
		*F*-_C × T_	1.922	0.123
				
	Yangmai 18	control	48.33a ± 2.08	42.94a ± 0.32
		2°C	46.33a ± 2.52	41.15b ± 0.06
		0°C	46.67a ± 1.53	40.86b ± 0.19
		−2°C	41.67b ± 1.53	38.89c ± 0.26
	Yannong 19	control	41.33a ± 0.58	38.89a ± 0.30
2016–2017		2°C	38.67a ± 2.08	36.46b ± 0.35
		0°C	39.33a ± 2.52	34.89c ± 0.30
		−2°C	32.33b ± 0.58	34.41c ± 0.10
	*F*-value	*F*-_Cultivar (C)_	98.648^∗∗^	1971.18^∗∗^
		*F*-_Treatment (T)_	18.309^∗∗^	276.745^∗∗^
		*F*-_C × T_	0.432	14.572^∗∗^

*Wheat plants were placed under 4°C for 60 h in the 2015–2016 growing season and at 2, 0, or −2°C for 24 h in the 2016–2017 growing season. F-_C × T_ refers to F-values of the interaction between cultivar and treatment. Data represent means ± SE (n = 3). Lowercase letters following data within the same column refer to significant differences (P < 0.05). ^∗∗^Significant differences at 0.01.*

**Table 4:** Correlation coefficients between yield factors, enzyme activities, and hormone contents under low temperature stress.

Cultivar	Yield factors	SC	SPS	SuS	Inv	ABA	IAA	GA
Yangmai 18	GW	−0.953^∗^	−0.949	0.133	0.967^∗^	−0.952^∗^	0.966^∗^	0.964^∗^
	GN	−0.975^∗^	−0.878	0.251	0.873	−0.969^∗^	0.867	0.841

Yannong 19	GW	−0.985^∗^	−0.844	0.176	0.916	−0.817	0.942	0.935
	GN	−0.842	−0.917	0.365	0.938	−0.953^∗^	0.920	0.870

*SC, sucrose content (mg/g FW); SPS, phosphosucrose synthase activity (U/mg pro); SuS, sucrose synthase activity (U/mg pro); Inv, invertase activity (U/mg pro); ABA, abscisic acid content (ng/g FW); IAA, indoleacetic acid content (ng/g FW); GA, gibberellic acid content (ng/g FW);GW, 1000-grain weight (g); GN, Grain number per ear. ^∗^Significant differences at 0.05, R_0.05_ = 0.950, R_0.01_ = 0.990.*

Sucrose phosphate synthase is a key enzyme of sucrose synthesis in plants. Accumulating evidence suggests that the ratio of sucrose to starch is positively correlated with SPS activity in the leaves (Ono et al., 1999). When plants are exposed to LT, drought or high salt stress, SPS activity tends to increase, thereby increasing the contents of soluble sugars such as sucrose and altering the osmotic pressure of the cells, allowing them to resist the stressful environment. It was previously revealed that LT treatment causes a significant increase in transcription levels of the SPS gene in kiwi fruit (Langenkämper et al., 1998). In this study, a similar increase in SPS activity in the wheat spikes was observed following LT stress at booting, and moreover, this increase increased with decreasing temperature. Except for treatment at 2°C for 24 h, the differences between treatments and controls were significant. In a previous study, LT stress caused a similar accumulation of sucrose in wheat ears, and this was positively correlated with the increase in SPS activity (Yang et al., 2001).

SuS and Inv are both implicated in the degradation of sucrose. SuS is responsible for catalyzing the reversible reaction of sucrose decomposition and synthesis as follows, sucrose + uridine diphosphate ↔ fructose + uridine diphosphate glucose (UDPG), playing a major role in catalyzing sucrose degradation. SuS can also catalyze the breakdown of sucrose into glucose and fructose during starch synthesis. In this study, SuS activity in the wheat ears increased significantly after treatment at 4°C for 60 h, possibly due to the effect of LT on sucrose accumulation, and with increasing SuS activity the decomposition of sucrose increased. Previous studies have also shown a significant increase in SuS decomposition activity under salt stress, LT and dehydration, when the demand for sugar decomposition increases (Römer et al., 2004; Haagenson et al., 2006; Klotz and Haagenson, 2008). Similarly, SuS activity was previously found to increase in wheat plants under LT stress and continued to increase with the accumulation of sucrose (Crespi et al., 1991; Gordon et al., 1999). In this study, decreasing temperature to −2°C for 24 h caused a decrease in SuS activity in spikelets of Yangmai 18 and Yannong 19. These findings suggest that the decrease in SuS activity under LT stress has a beneficial effect on sucrose accumulation and cold resistance in wheat, with a sacrifice to overall growth. In rice, LT at booting caused a decrease in the activity of sucrose-degrading enzyme in the anthers, with downregulation of monosaccharide transporter expression, resulting in insufficient sucrose supply to the tapetum and pollen grains and subsequent pollen abortion (Oliver et al., 2005, 2007).

Under LT stress, the apoplast transport pathway involved in Inv and monosaccharide protein transport is inhibited, affecting the transport of sucrose to the anthers (Sherson et al., 2003). Pollen sterility therefore occurs due to an increase in cell wall-bound AI activity, inhibiting the supply of sucrose, and limiting and disrupting pollen development (Oliver et al., 2005; Gothandam et al., 2007). As a result, microspore development is hindered because of the reduced levels of glucose, sucrose and fructose, thereby resulting in infertile pollen (Arshad et al., 2017). In this study, AI activity in the wheat ears decreased after LT treatment, and this decrease increased with decreasing temperature. AI activity also differed between spikelets, with significant differences in the US and LS under all temperature treatments. Meanwhile, the decrease in AI activity in the MS was relatively lower, suggesting that AI activity in the MS is less affected by LT stress, facilitating sucrose transport to the pollen.

Although the changes in SPS, SuS and, Inv activity in relation to sucrose metabolism under cold stress are not completely consistent between studies, our findings suggest that wheat ears accumulate a large amount of sucrose during adaptation to adapt to LT stress. Activity of the main enzymes involved in sucrose metabolism was significantly affected by the variety, temperature treatment and spikelet location. These findings suggest that alterations in activity of these enzymes affect the decomposition and transport of sucrose, leading to abnormal development of florets.

### Effects of LT at Booting on the Expression of Genes Involved in Sucrose Metabolism

Inhibition of sucrose degradation leads to failure of plant reproductive function under abiotic stress (Ruan, 2014). In line with this, Zeng et al. (2011) revealed that LT stress leads to upregulation of the genes involved in sugar synthesis, metabolism and transport. Cold treatment in sugar beet caused upregulation of the sucrose synthase gene, and expression was twice that of the control group after 7 days of treatment (Klotz and Haagenson, 2008). Moreover, transcriptional levels of sucrose synthase in alfalfa were found to decrease under LT (Bertrand et al., 2017). LT stress was also found to cause differential changes in the expression of sucrose synthase genes in different winter wheat organs. Expression was found to decrease gradually in tillering nodes, while in leaves, an initial increase followed by a decrease was observed (Zeng et al., 2011). Activity of the main enzymes involved in sucrose metabolism was significantly affected by the variety, temperature treatment, and spikelet location.

In monocotyledonous plants, SuS is encoded by two non-allele genes, *SUS1* and *SUS2*. In this study, expression of *TaSUS1* was upregulated, while expression of *TaSUS2* was reduced in wheat ears following treatment at −2°C for 24 h. The change in SuS activity suggests that *TaSUS1* and *TaSUS2* co-regulate SuS activity under LT stress at booting. The findings also suggest that *TaSUS2* plays a greater role in the response to LT stress. Abiotic stress inhibits photosynthesis, reducing sucrose transfer and leading to inhibition of Inv gene expression (Boyer and McLaughlin, 2007). Moreover, there is convincing evidence that male sterility and resulting grain or fruit abortion occur as a result of reduced expression of cell wall invertase and vacuolar invertase in anthers and pollen of wheat under drought conditions (Koonjul et al., 2005). In this study, expression of *TaCWI* in wheat ears was significantly downregulated after −2°C treatment for 24 h, especially in the cold-sensitive variety Yangmai 18.

### Effects of LT Stress on Endogenous Hormone Contents

Phytohormones are important growth regulators, playing important roles in regulating balance and inducing stress resistance in plants under LT and other stress conditions. Increased endogenous ABA levels can induce expression of stress genes and enhance cold tolerance (Yang et al., 2011). Moreover, Lalk and Dorffling (1985) revealed an increase in ABA content during cold acclimation in winter wheat, inducing expression of stress genes and improving cold tolerance. In this study, a similar increase in ABA content was observed following LT stress. Significant inhibition of invertase gene expression in the spikes was previously observed under ABA treatment, affecting sugar metabolism and the source-sink relationship in the panicles (Ji et al., 2011). In general, an increase in ABA content under LT stress might amplify ABA signaling and initiate changes in the expression of low-temperature-related genes in downstream ABA responses, thereby enhancing tolerance to cold. However, plant adaptation to stress and growth involves two conflicting processes, adaptation to adversity, which negatively affects growth, and vice versa (Scheres and van der Putten, 2017). Under persistent LT stress, plants need to maintain balance between normal growth and adaptive stress to avoid over-amplification of stress signals (Zong et al., 2016). Maintaining a relatively low level of ABA under LT stress within a certain threshold range is therefore conducive to increasing tolerance to LT. In this study, the ABA content of young ears of the cold-tolerant variety Yannong 19 was lower than that of the cold-sensitive cultivar Yangmai 18, suggesting that Yannong 19 is able to keep growing under such stress conditions.

Growth delay is a common phenomenon in plants during adaptation to environmental stress (Scottin et al., 2004). Inhibition of the endogenous growth-promoting GA signaling pathway significantly increases plant survival under stress (Achard et al., 2006). Moreover, under LT stress, the content of IAA in *Davidia involucrata* and wheat were found to decrease with decreasing temperature (Kosová et al., 2010). LT treatment of tobacco also caused a significant decrease in the active gibberellin GA_1_ content (Niu et al., 2013). LT at booting also caused a reduction in the endogenous GA content of rice, while GA treatment restored pollen fertility of the GA synthesis mutant under LT stress, thereby restoring the seed setting rate (Sakata et al., 2014). In this study, the contents of IAA and GA decreased significantly under LT stress at booting, resulting in insufficient nutrient input. Moreover, the decrease was greater in the US and LS than the MS, suggesting that the late-developing upper and lower florets are more affected by LT stress. Overall, IAA and GA contents in young ears of the cold-sensitive cultivar Yangmai 18 were lower than those of the cold-tolerant cultivar Yannong 19 under each temperature treatment, suggesting that growth inhibition was more severe in Yangmai 18 than Yannong 19.

### Relationships Among Sucrose Metabolism, Endogenous Hormone Content and Yield Under LT Stress

At booting, wheat is very sensitive to LT stress, resulting in a large reduction in pollen mother cells and pollen, and subsequently, a serious decline in yield (Dolferus et al., 2011; Barton et al., 2014). Early temperature stress was previously found to have a significant effect on the final grain weight, with larger negative effects observed when temperature stress was close to the anthesis (Calderini et al., 2001; Ugarte et al., 2007). The results of this study suggests that LT stress at booting caused a decrease in the grain number per ear and 1000-grain weight, most notably the latter. LT stress reduces the supply of soluble sugars to reproductive tissue (Nayyar et al., 2005). Endogenous hormones are used as a signal during the LT stress response, affecting sucrose metabolism in the spikes by regulating expression of the genes related to sucrose metabolism-related enzymes (Ji et al., 2011). Analysis of the correlation between sucrose metabolism-related enzyme activity, hormone content and yield (1000-grain weight and grain number per ear) revealed a negative correlation between sucrose content, SPS activity, ABA content and yield. Moreover, the correlation between sucrose content, ABA content and yield was significant in Yangmai 18. The correlation coefficient between SuS activity and yield (1000-grain weight, grain number per ear) was lower than that between SPS activity, Inv activity and yield. The results also revealed that the changes in SuS activity and gene expression were complicated by LT stress, suggesting that the response of SuS is more complex and requires further study. The correlation coefficients between sucrose metabolizing enzyme activity, hormone content and 1000-grain weight were higher in cold-sensitive variety Yangmai 18 than cold-tolerant cultivar Yannong 19. Comparisons between the two varieties revealed significant positive correlations between 1000-grain weight and Inv, IAA, GA in Yangmai 18 but not Yannong 19. These findings suggest that under LT stress, the response of the cold-sensitive variety is understandably more affected, having a greater effect on growth and development of the spikes. However, the response in the cold-tolerant variety was relatively slow, and the negative effects of hormone and enzyme activity on yield were relatively weak.

## Conclusion

Our findings suggest that LT stress at booting delays the development of wheat spikelets and florets by increasing the sucrose content of the young ears and altering sucrose metabolism-related enzyme activity and gene expression. Activity of SPS was enhanced, while that of Inv and SuS were reduced, and expression of *CWINV* was inhibited. Moreover, the contents of IAA and GA, which promote the growth of young ears, decreased, while that of ABA, which hinders growth, increased. In addition, even after only a short period of LT stress at booting, the number of spikelets and 1000-grain weight decreased as a result of these changes in hormone content and sucrose metabolism. In conclusion, therefore, LT stress at booting alters the hormone content of young spikes, affecting the transport and metabolism of sucrose and hindering spikelet development, thereby decreasing yield.

## Author Contributions

WZ and ZH designed the experiments. WZ conducted the study and prepared the manuscript. JW, LM, and KX collected and analyzed the data, and helped in sampling and the physiological parameter measurements. YF and SM helped in drafting the manuscript. DJ assisted in manuscript editing. All authors read and approved the final manuscript.

## Conflict of Interest Statement

The authors declare that the research was conducted in the absence of any commercial or financial relationships that could be construed as a potential conflict of interest.

## References

[B1] Achard P. Cheng H. De Grauwe L. Decat J. Schoutteten H. Moritz T. (2006). Integration of plant responses to environmentally activated phytohormonal signals. *Science* 311 91–94. 10.1126/science.1118642 16400150

[B2] Arshad M. S. Farooq M. Asch F. Krishna J. S. V. Prasad P. V. V. Siddique K. H. M. (2017). Thermal stress impacts reproductive development and grain yield in rice. *Plant Physiol. Bioch.* 115 57–72. 10.1016/j.plaphy.2017.03.011 28324683

[B3] Barton D. A. Cantrill L. C. Law A. M. K. Phillips C. G. Sutton B. G. Overall R. L. (2014). Chilling to zero degrees disrupts pollen formation but not meiotic microtubule arrays in *Triticum aestivum* L. *Plant Cell Environ.* 37 2781–2794. 10.1111/pce.12358 24762030

[B4] Bertrand A. Bipfubusa M. Claessens A. Rocher S. Castonguay Y. (2017). Effect of photoperiod prior to cold acclimation on freezing tolerance and carbohydrate metabolism in alfalfa (*Medicago sativa* L.). *Plant Sci.* 264 122–128. 10.1016/j.plantsci.2017.09.003 28969792

[B5] Boyer J. S. McLaughlin J. E. (2007). Functional reversion to identify controlling genes in multigenic responses: analysis of floral abortion. *J. Exp. Bot.* 58 267–277. 10.1093/jxb/erl177 17105969

[B6] Calderini D. F. Savin R. Abeledo L. G. Reynolds M. P. Slafer G. A. (2001). The importance of the period immediately preceding anthesis for grain weight determination in wheat. *Euphytica* 119 199–204. 10.1023/A:101759792

[B7] Castro A. J. Clément C. (2007). Sucrose and starch catabolism in the anther of Lilium during its development: a comparative study among the anther wall, locular fluid and microspore/pollen fractions. *Planta* 225 1573–1582. 10.1007/s00425-006-0443-5 17123100

[B8] Crespi M. D. Zabaleta E. J. Pontis H. G. Salermo G. (1991). Sucrose synthase expression during cold acclimation in wheat. *Plant physiol.* 96 887–891. 10.1104/pp.96.3.887 16668270 PMC1080860

[B9] Dolferus R. Ji X. Richards R. A. (2011). Abiotic stress and control of grain number in cereals. *Plant Sci.* 181 331–341. 10.1016/j.plantsci.2011.05.015 21889038

[B10] Gordon A. J. Minchin F. R. James C. L. Komina O. (1999). Sucrose synthase in legume nodules is essential for nitrogen fixation. *Plant Physiol.* 120 867–878. 10.1104/pp.120.3.867 10398723 PMC59326

[B11] Gothandam K. M. Kim E. S. Chung Y. Y. (2007). Ultrastructural study of rice tapetum under low-temperature stress. *J. Plant Biol.* 50 396–402. 10.1007/BF03030674

[B12] Haagenson D. M. Klotz K. L. Mcgrath J. M. (2006). Sugar beet sucrose synthase genes differ in organ-specific and developmental expression. *J. Plant Physiol.* 163 102–106. 10.1016/j.jplph.2005.05.006 16360809

[B13] Han H. Tian Z. Fan Y. Cui Y. Cai J. Jiang D. (2015). Water-deficit treatment followed by re-watering stimulates seminal root growth associated with hormone balance and photosynthesis in wheat (*Triticum aestivum* L.) seedlings. *Plant Growth Regul.* 77 201–210. 10.1007/s10725-015-0053-y

[B14] Hendrix D. L. (1993). Rapid extraction and analysis of nonstructural carbohydrates in plant tissues. *Crop Sci.* 33 1306–1311. 10.2135/cropsci1993.0011183X003300060037x

[B15] Ji C. H. Li H. Y. Chen L. B. Xie M. Wang F. P. Chen Y. L. (2013). A novel rice bHLH transcription factor. DTD, acts coordinately with TDR in controlling tapetum function and pollen development. *Mol. Plant* 6 1715–1718. 10.1093/mp/sst046 23519457

[B16] Ji X. M. Dong B. D. Shiran B. Talbot M. J. Edlington J. E. Trijntje H. (2011). Control of ABA catabolism and ABA homeostasis is important for reproductive stage stress tolerance in cereals. *Plant Physiol.* 156 647–662. 10.1104/pp.111.176164 21502188 PMC3177265

[B17] Klotz K. L. Haagenson D. M. (2008). Wounding, anoxia and cold induce sugar beet sucrose synthase transcriptional changes that are unrelated to protein expression and activity. *J. Plant Physiol.* 165 423–434. 10.1016/j.jplph.2007.02.001 17395334

[B18] Koonjul P. K. Minhas J. S. Nunes C. Sheoran I. S. Saini H. S. (2005). Selective transcriptional down-regulation of anther invertases precedes the failure of pollen development in water-stressed wheat. *J. Exp. Bot.* 56 179–190. 10.1093/jxb/eri018 15533880

[B19] Kosová K. Prášil I. T. Vítámvás P. Dobrev P. Motyka V. Floková K. (2010). Complex phytohormone responses during the cold acclimation of two wheat cultivars differing in cold tolerance, winter Samanta and spring Sandra. *J. Plant Physiol.* 169 567–576. 10.1016/j.jplph.2011.12.013 22304971

[B20] Lalk I. Dorffling K. (1985). Hardening abscisic-acid proline and freezing resistance in two winter wheat triticum-aestivum cultivars. *Physiol. Plant.* 63 287–292. 10.1111/j.1399-3054.1985.tb04267.x

[B21] Langenkämper G. McHale R. Gardner R. C. MacRae E. (1998). Sucrose-phosphate synthase steady-state mRNA increases in ripening kiwifruit. *Plant Mol. Biol.* 36 857–869. 10.1023/A:1005964812161 9520277

[B22] Li X. Jiang D. Liu F. (2016). Winter soil warming exacerbates the impacts of spring low temperature stress on wheat. *J. Agron. Crop Sci.* 202 554–563. 10.1111/jac.12177

[B23] Li X. N. Cai J. Liu F. L. Dai T. B. Cao W. X. Jiang D. (2014). Physiological, proteomic and transcriptional responses of wheat to combination of drought or waterlogging with late spring low temperature. *Funct. Plant Biol.* 41 690–703. 10.1071/FP13306 32481024

[B24] Livak K. J. Schmittgen T. D. (2001). Analysis of relative gene expression data using real-time quantitative PCR and the 2-ΔΔCT method. *Methods* 25 402–408. 10.1006/meth.2001.1262 11846609

[B25] Mittler R. Blumwald E. (2015). The roles of ROS and ABA in systemic acquired acclimation. *Plant Cell* 27 64–70. 10.1105/tpc.114.133090 25604442 PMC4330577

[B26] Nayyar H. Bains T. S. Kumar S. Kaur G. (2005). Chilling effects during seed filling on accumulation of seed reserves and yield of chickpea. *J. Sci. Food Agric.* 85 1925–1930. 10.1002/jsfa.2198

[B27] Niu N. N. Liang W. Q. Yang X. J. Jin W. L. Wilson Z. A. Hu J. P. (2013). EAT1 promotes tapetal cell death by regulating aspartic proteases during male reproductive development in rice. *Nat. Commun.* 4:1445. 10.1038/ncomms2396 23385589

[B28] Oliver S. N. Dennis E. S. Dolferus R. (2007). ABA regulates apoplastic sugar transport and is a potential signal for cold-induced pollen sterility in rice. *Plant Cell Physiol.* 48 1319–1330. 10.1093/pep/pcm100 17693452

[B29] Oliver S. N. Van Dongen J. T. Alfred S. C. Mamun E. A. Zhao X. C. Saini H. S. (2005). Cold-induced repression of the rice anther-specific cell wall invertase gene OSINV4 is correlated with sucrose accumulation and pollen sterility. *Plant Cell Environ.* 28 1534–1551. 10.1111/j.1365-3040.2005.01390.x

[B30] Ono K. Ishimaru K. Aoki N. Takahashi S. Ozawa K. Ohkawa Y. (1999). Characterization of a maize sucrose-phosphate synthase protein and its effect on carbon partitioning in transgenic rice plants. *Plant Prod. Sci.* 2 172–177. 10.1626/pps.2.172

[B31] Römer U. Schrader H. Günther N. Nettelstroth N. Frommer W. B. Elling L. (2004). Expression, purification and characterization of recombinant sucrose synthase 1 from *Solanum tuberosum* L. for carbohydrate engineering. *J. Biotechnol.* 107 135–149. 10.1016/j.jbiotec.2003.10.017 14711497

[B32] Ruan Y. L. (2014). Sucrose metabolism: gateway to diverse carbon use and sugar signaling. *Annu. Rev. Plant Biol.* 65 33–67. 10.1146/annurev-arplant-050213-040251 24579990

[B33] Sakata T. Oda S. Tsunaga Y. Shomura H. Kawagishi-Kobayashi M. Aya K. (2014). Reduction of gibberellin by low temperature disrupts pollen development in rice. *Plant Physiol.* 164 2011–2019. 10.1104/pp.113.234401 24569847 PMC3982758

[B34] Scheres B. van der Putten W. H. (2017). The plant perceptron connects environment to development. *Nature* 543 337–345. 10.1038/nature22010 28300110

[B35] Scottin I. M. Clarke S. M. Wood J. E. Mur L. A. J. (2004). Salicylate accumulation inhibits growth at chilling temperature in *Arabidopsis*. *Plant Physiol.* 135 1040–1049. 10.1104/pp.104.041293 15173571 PMC514138

[B36] Serrago A. R. Miralles D. J. Slafer G. A. (2008). Floret fertility in wheat as affected by photoperiod during stem elongation and removal of spikelets at booting. *Eur. J. Agron.* 28 301–308. 10.1016/j.eja.2007.08.004

[B37] Sharma S. Sreenivasulu N. Harshavardhan V. T. Seiler C. Sharma S. Khalil Z. N. (2010). Delineating the structural, functional and evolutionary relationships of sucrose phosphate synthase gene family II in wheat and related grasses. *BMC Plant Biol.* 10:134. 10.1186/1471-2229-10-134 20591144 PMC3017794

[B38] Sherson S. M. Alford H. L. Forbes S. M. Wallace G. Smith S. M. (2003). Roles of cell-wall invertases and monosaccharide transporters in the growth and development of *Arabidopsis*. *J. Exp. Bot.* 54 525–531. 10.1093/jxb/erg055 12508063

[B39] Shu H. M. Zhou Z. G. Xu N. Y. Wang Y. H. Zheng M. (2009). Sucrose metabolism in cotton (*Gossypium hirsutum* L.) fibre under low temperature during fibre development. *Eur. J. Agron.* 31 61–68. 10.1016/j.eja.2009.03.004

[B40] Slafer G. A. Abeledo L. G. Miralles D. J. Gonzalez F. G. Whitechurch E. M. (2001). Photoperiod sensitivity during stem elongation as an avenue to raise potential yield in wheat. *Euphytica* 119 191–197. 10.1023/A:1017535632171

[B41] Sun X. C. Hu C. X. Tan Q. L. Liu J. S. Liu H. E. (2009). Effects of molybdenum on expression of cold-responsive genes in abscisic acid (ABA)-dependent and ABA-independent pathways in winter wheat under low-temperature stress. *Ann. Bot.* 104 345–356. 10.1093/aob/mcp133 19491090 PMC2710908

[B42] Tang R. S. Zheng J. C. Jin Z. Q. Zhang D. D. Huang Y. H. Chen L. G. (2008). Possible correlation between high temperature-induced floret sterility and endogenous levels of IAA, GA3 and ABA in rice (*Oryza sativa* L.). *Plant Growth Regul.* 54 37–43. 10.1007/s10725-007-9225-8

[B43] Thakura P. Kumara S. Malika J. A. Bergerb J. D. Nayyara H. (2010). Cold stress effects on reproductive development in grain crops: an overview. *Environ. Exp. Bot.* 67 429–443. 10.1016/j.envexpbot.2009.09.004

[B44] Ugarte C. Calderini D. F. Slafer G. A. (2007). Grain weight and grain number responsiveness to pre-anthesis temperature in wheat, barley and triticale. *Field Crops Res.* 100 240–248. 10.1016/j.fcr.2006.07.010

[B45] Wang J. Lin X. Sun Q. Jena K. K. (2013). Evaluation of cold tolerance for japonica rice varieties from different country. *Adv. J. Food Sci. Technol.* 5 54–56. 10.19026/ajfst.5.3311

[B46] Wanner L. A. Junttila O. (1999). Cold-induced freezing tolerance in *Arabidopsis*. *Plant Physiol.* 120 391–400. 10.1104/pp.120.2.391 10364390 PMC59277

[B47] Yang J. C. Zhang J. H. Wang Z. Q. Zhu Q. S. (2001). Activities of starch hydrolytic enzymes and sucrose-phosphate synthase in the stems of rice subjected to water stress during grain filling. *J. Exp. Bot.* 52 2169–2179. 10.1093/jexbot/52.364.2169 11604456

[B48] Yang W. Liu X. D. Chi X. J. Wu C. A. Li Y. Z. Song L. L. (2011). Dwarf apple MbDREB1 enhances plant tolerance to low temperature, drought, and salt stress via both ABA-dependent and ABA-independent pathways. *Planta* 233 219–229. 10.1007/s00425-010-1279-6 20967459

[B49] Zeng Y. Yu J. Cang J. Liu L. J. Mu Y. C. Wang J. H. (2011). Detection of sugar accumulation and expression levels of correlative key enzymes in winter wheat (*Triticum aestivum*) at low temperatures. *Biosci. Biotech. Biochem.* 75 681–687. 10.1271/bbb.100813 21512254

[B50] Zhang B. Jia D. Gao Z. Q. Dong Q. He L. H. (2016). Physiological responses to low temperature in spring and winter wheat varieties. *J. Sci. Food Agric.* 96 1967–1973. 10.1002/jsfa.7306 26095741

[B51] Zhang W. J. Huang Z. L. Wang Q. Guan Y. N. (2015). Effects of low temperature on leaf anatomy and photosynthetic performance in different genotypes of wheat following a rice crop. *Int. J. Agric. Biol.* 17 1165–1171. 10.17957/IJAB/15.0035

[B52] Zhang W. J. Shu H. M. Hu H. B. Chen B. L. Wang Y. H. Zhou Z. G. (2009). Genotypic differences in some physiological characteristics during cotton fiber thickening and its influence on fiber strength. *Acta Physiol. Plant.* 31 927–935. 10.1007/s11738-009-0306-3

[B53] Zhang X. F. Zheng Y. F. Wang C. Y. Chen H. L. Ren Z. H. Zou C. H. (2011). Spatial distribution and temporal variation of the winter wheat late frost disaster in Henan, China. *Acta Meteorol. Sin.* 25 249–259. 10.1007/s13351-011-0031-x

[B54] Zong W. Tang N. Yang J. Peng L. Ma S. Q. Xu Y. (2016). Feedback regulation of ABA signaling and biosynthesis by a bZIP transcription factor targets drought-resistance-related genes. *Plant Physiol.* 171 2810–2825. 10.1104/pp.16.00469 27325665 PMC4972276

